# The Functional Outcome of Proximal Femoral Nail Antirotation-2 (PFNA-2) in Peritrochanteric Fractures of the Femur

**DOI:** 10.7759/cureus.64839

**Published:** 2024-07-18

**Authors:** Gagandeep Singh, Anoop Kumar, Mohhamad Farooq Bhat, Amit Thakur

**Affiliations:** 1 Orthopedics, All India Institute of Medical Sciences, Vijaypur, Jammu, IND; 2 Orthopedics, Government Medical College and Hospital, Jammu, IND

**Keywords:** pfn-a2, modified harris hip score, peritrochanteric fractures, subtrochanteric fractures, intertrochanteric fractures

## Abstract

Introduction

Peritrochanteric fractures are defined as extra-articular fractures involving the trochanter and frequent extension into the subtrochanteric region. These fractures exhibit a bimodal distribution in terms of age. These fractures commonly happen in young individuals who experience high-energy trauma, often in combination with other injuries. In contrast, elderly individuals with osteopenia are more prone to fractures caused by low-energy trauma.

Methods

This study is a prospective investigation that was carried out over 30 months. The study focused on peritrochanteric fractures that were treated using proximal femoral nail antirotation-2 (PFNA-2) as a fixation device. A range of criteria were examined and documented, encompassing the mean duration of surgical procedures, blood loss, the duration of hospitalization, mobility, and any potential post-operative problems. Subsequent assessments were conducted at fixed intervals of two weeks, six weeks, three months, six months, and two years. The functional outcome analysis for all patients involved the utilization of the modified Harris hip score (HHS).

Results

The study involved 60 cases of peritrochanteric fractures. The average age was 56 years. The most common mode of trauma was trivial fall/slip (46.66%), followed by road traffic accidents (RTA) (31.66%) and falls from height (21.66%). The average operating time was 53.03±5.66 minutes. The average modified Harris hip score was 84.78 with 26.66% excellent, 70% good, and 3.33% fair results. Complications included superficial wound infection (5%), knee stiffness (11.66%), hip pain (8.33%), and shortening (1.66%).

Conclusion

PFNA-2 is a safe and easy implant option for treating peritrochanteric fractures as it preserves periosteal covering, minimizes blood loss, has a short operative time, and helps in early mobilization. PFNA-2 provides excellent outcomes in patients with peritrochanteric fractures with minimum complication rates compared to all other open methods and is highly recommendable.

## Introduction

Peritrochanteric fractures are characterized as fractures that occur outside the joint and primarily affect the trochanter, often extending into the subtrochanteric region. Therefore, these fractures can be classified into two distinct categories: intertrochanteric fractures and subtrochanteric fractures. The fractures have a bimodal distribution in terms of age. These fractures occur in young individuals as a result of severe and forceful trauma. In individuals of advanced age suffering from osteoporosis, these fractures frequently occur as a result of a fall from a standing position with minimal force applied [1]. Fractures in younger individuals often result from high-energy trauma, such as car accidents or falls from height [2]. Fragility fractures in the trochanteric region in the elderly are often caused by low-energy traumas such as falls [3]. In the preceding half-century, a diverse range of implants and fastening techniques have been employed for the purpose of surgical stabilization. Implementing enhanced sterilization methods and utilizing modular theatres with laminar flow have significantly decreased infection rates. Advancements in biology, reduction techniques, and biomechanically enhanced implants have significantly improved the treatment of peritrochanteric fractures. There exist multiple internal fixing alternatives for the management of these fractures, which can be broadly classified into two categories: intramedullary fixation and plating. The aim of this study was to assess the functional outcome of proximal femoral nail antirotation-2 (PFNA-2) (intramedullary implant) in peritrochanteric fracture fixation.

## Materials and methods

This study is a prospective investigation carried out between November 2019 and March 2022. All participants in the study provided written informed consent prior to their involvement. The study focused on peritrochanteric fractures that were treated using proximal femoral nail antirotation-2 (PFNA-2) as a fixation device. A range of criteria were examined and documented, encompassing the mean duration of surgical procedures, blood loss, the duration of hospitalization, and any potential post-operative problems. The study was approved by the Government Medical College, Jammu, Institutional Ethics Committee (approval number: IEC/GMC/2022/798).

Inclusion criteria

The study included individuals who were more than 18 years old, individuals who had radiological findings confirming peritrochanteric fracture, and patients who were medically fit and willing for surgery.

Exclusion criteria

The study excluded individuals who had compound fractures and patients who had pathological fractures.

Operative technique

The surgical procedure was performed using spinal or general anesthesia. Prior to surgery, the patient received a single intravenous dosage of broad-spectrum cephalosporin. In all instances, a fracture table and image intensifier were employed. A reduction was performed on the fracture using the C-arm. Under all aseptic precautions, the part was prepared and draped. Fracture reduction was checked under C-arm, and satisfactory reduction was achieved via closed or mini open method. In some cases, K-wires were used to hold the reduction in place. A skin incision was given, and entry was made with awl under the guidance of the C-arm; guide wire was inserted, followed by serial reaming of the medullary canal; the PFNA-2 was inserted. The guide wire was passed into the femoral neck in a manner that positioned the blade in the lower portion of the neck in the anteroposterior view and centrally in the lateral view. A hammer was used to introduce the helical blade, which was attached to a specific inserter over the guide wire. The distal locking of the PFNA-2 was achieved using either dynamic or static means.

Post-operative protocol

The patients' limb was raised on a pillow, and they were closely monitored in the recovery room until they were stable, after which they were sent to the ward. Intravenous antibiotics were administered for the initial 48 hours, followed by a transition to oral administration for a duration of three days. A post-operative elastic capillary bandage was employed to prevent deep vein thrombosis (DVT) in elderly individuals (above 60 years). The initiation of static quadriceps exercises was done on the first day following the surgical procedure. On the following day of the surgical procedure, active quadriceps and hip flexion exercises were initiated. The post-operative dressing was done on the second and fifth days. The sutures were removed on the 14th day after the surgery. Partial weight-bearing walking was initiated between three days and six weeks after surgery, depending on the specific fracture pattern. Following the evaluation of the radiological and clinical union, complete weight-bearing walking was permitted. All patients started full weight-bearing by eight weeks post-operatively. The participants were called for periodic follow-up appointments at intervals of two weeks, six weeks, three months, six months, and two years. The functional outcome analysis for all patients was done using the modified Harris hip score (HHS).

## Results

The research encompassed a total of 67 verified cases of peritrochanteric fractures, regardless of gender. Among the total of 67 patients, six cases were lost to follow-up, while one case expired owing to causes that were not related to the surgical procedure. The subsequent study pertains to the data of the remaining 60 patients, encompassing both intraoperative data and post-operative outcomes. Various parameters studied are detailed in Table 1. The age range of the participants spanned from 20 to 102 years. The mean age of the participants was 56.56±19.34 years. The majority of the patients, comprising 11 individuals (18.33%), were within the age range of 60-69 years. In our study, 37 patients (61.66%) had left-sided fractures, and 23 patients (38.33%) had right-sided fractures. Our study had 41 male (68.88%) and 19 female (31.66%) patients. Males (68.88%) had a relatively higher percentage of fractures as compared to females (31.66%), with a male-to-female ratio of 2.15:1. The primary causes of injury were minor falls or slips below a height of 6 ft, falls from heights greater than 6 ft, and road traffic accidents (RTA). The majority of the patients who experienced a minor fall were of advanced age and had osteoporosis.

**Table 1:** Various parameters in the present study RTA, road traffic accidents; HBsAg, hepatitis B surface antigen; HCV, hepatitis C virus

Serial number	Variable		Number of patients (n) along with percentage
1.	Mean age	56.56±19.34 years	
2.	Side	Right	23 (38.33%)
		Left	37 (61.66%)
3.	Gender	Male	41 (68.88%)
		Female	19 (31.66%)
4.	Mode of injury	Fall from height	13 (21.66%)
		Trivial fall	28 (46.66%)
		RTA	19 (31.66%)
5.	Fracture pattern (intertrochanteric fractures are categorized according to Boyd and Griffin's (BG) classification, while subtrochanteric fractures are classified according to Seinsheimer's classification)	BG type 1	0
		BG type 2	24 (40%)
		BG type 3	2 (3.33%)
		BG type 4	13 (21.66%)
		Type I	0
		Type IIA	3 (5%)
		Type IIB	7 (11.66%)
		Type IIC	2 (3.33%)
		Type IIIA	5 (8.33%)
		Type IIIB	3 (5%)
		Type IV	1 (1.66%)
6.	Method of reduction	Closed	52 (86.66%)
		Open	8 (13.33%)
7.	Comorbidities	Diabetes	11 (18.33%)
		Hypertension	9 (15%)
		Hypothyroidism	1 (1.66%)
		HBsAg	1 (1.66%)
		HCV	1 (1.66%)
8.	Complications	Knee stiffness	7 (11.66%)
		Superficial infection	6 (10%)
		Hip pain	5 (8.33%)
		Shortening	1 (1.66%)
9.	Modified Harris hip score	Excellent	16 (26.66%)
		Good	42 (70%)
		Fair	2 (3.33%)
		Poor	0
10.	Average modified Harris hip score	84.78±4.31	

**Figure 1:**
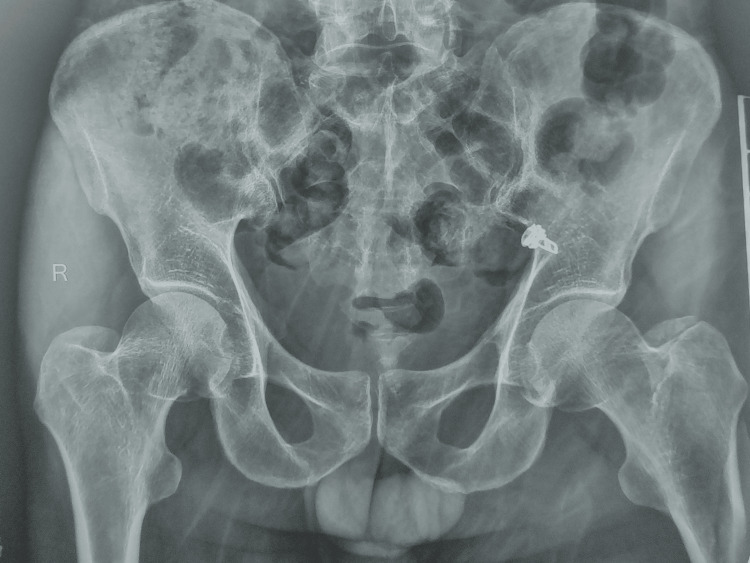
Preoperative X-ray (AP view) showing the pelvis with both hips AP: anteroposterior

**Figure 2:**
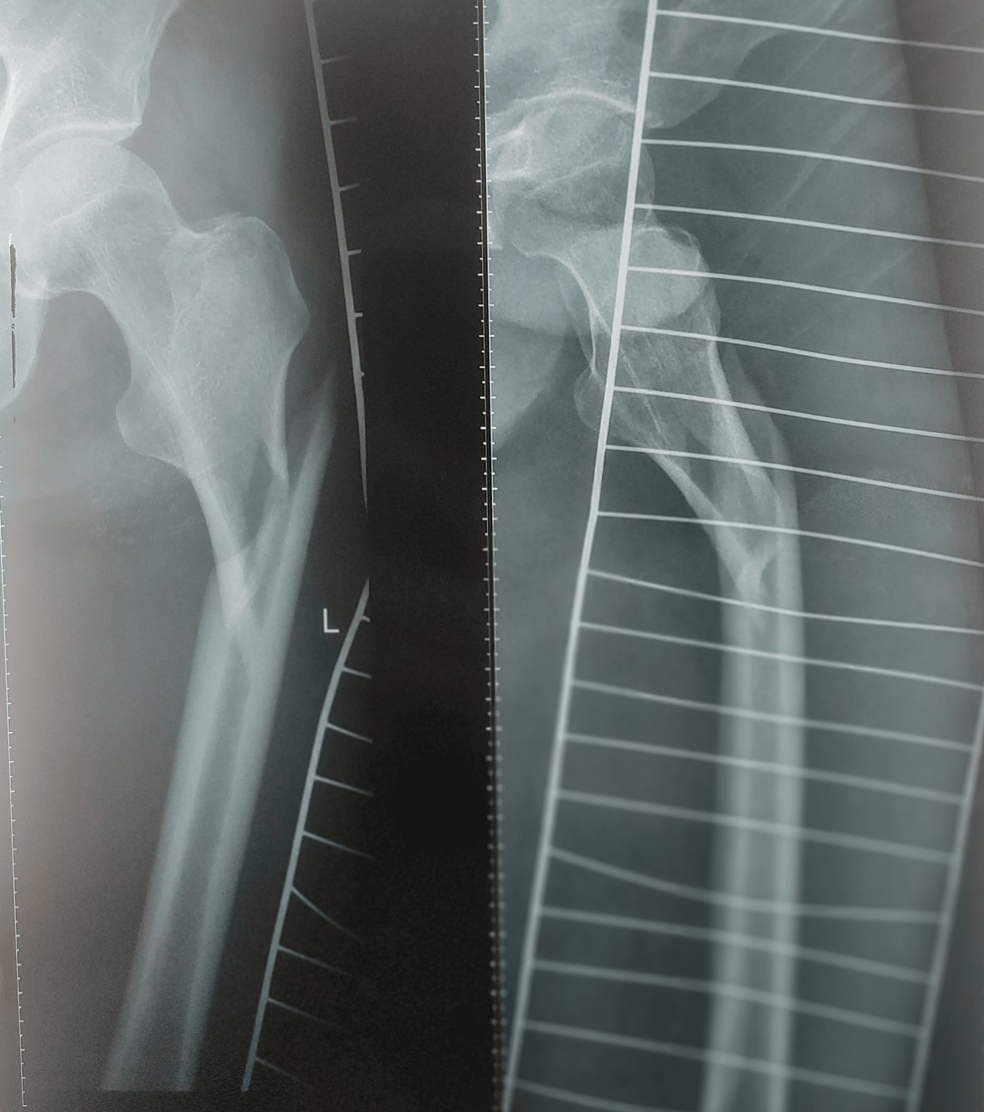
Preoperative X-ray (lateral view) showing the subtrochanteric fracture

**Figure 3:**
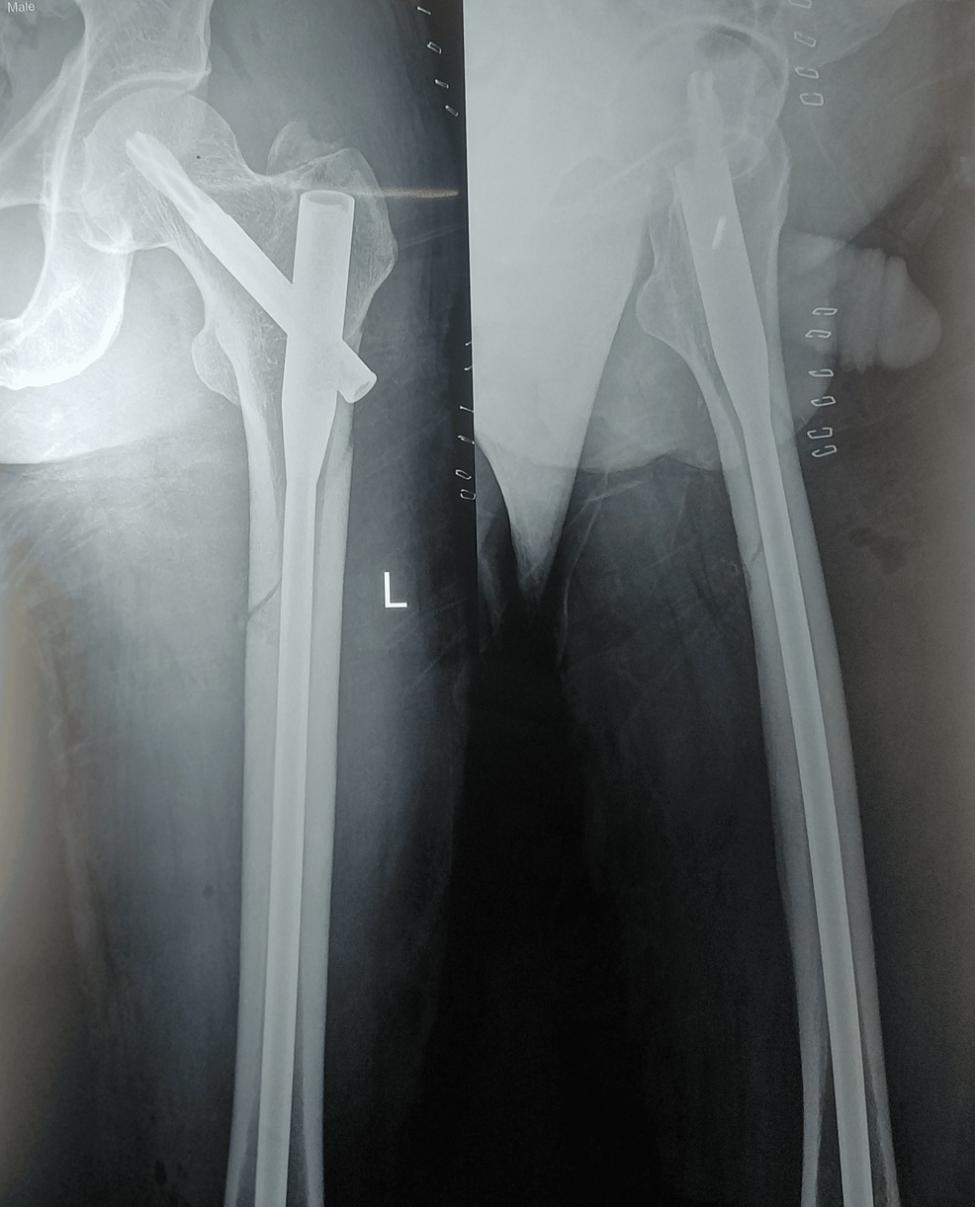
Post-operative X-ray (AP view) showing subtrochanteric fracture fixed with long PFNA-2 PFNA-2, proximal femoral nail antirotation-2; AP, anteroposterior

**Figure 4:**
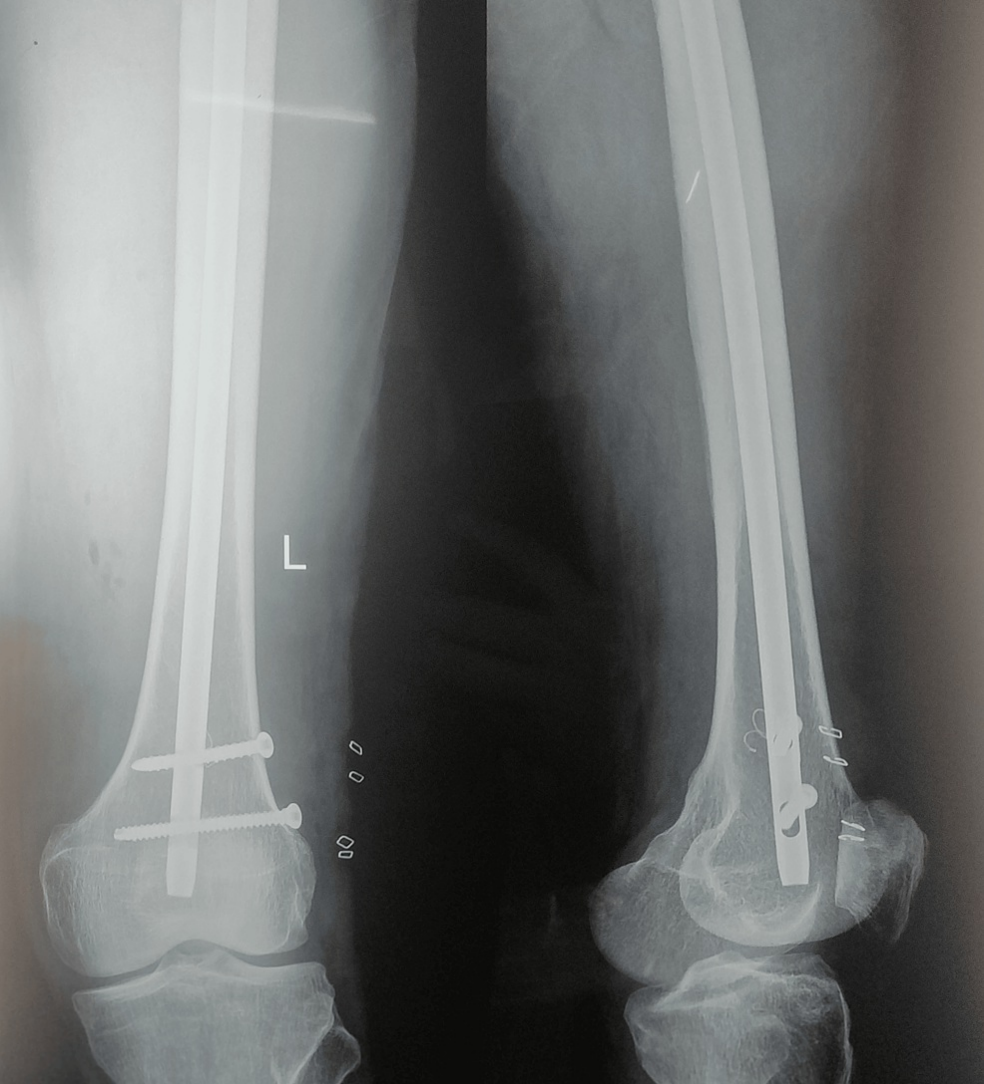
Post-operative X-ray (AP view) showing subtrochanteric fracture fixed with long PFNA-2 PFNA-2, proximal femoral nail antirotation-2; AP, anteroposterior

**Figure 5:**
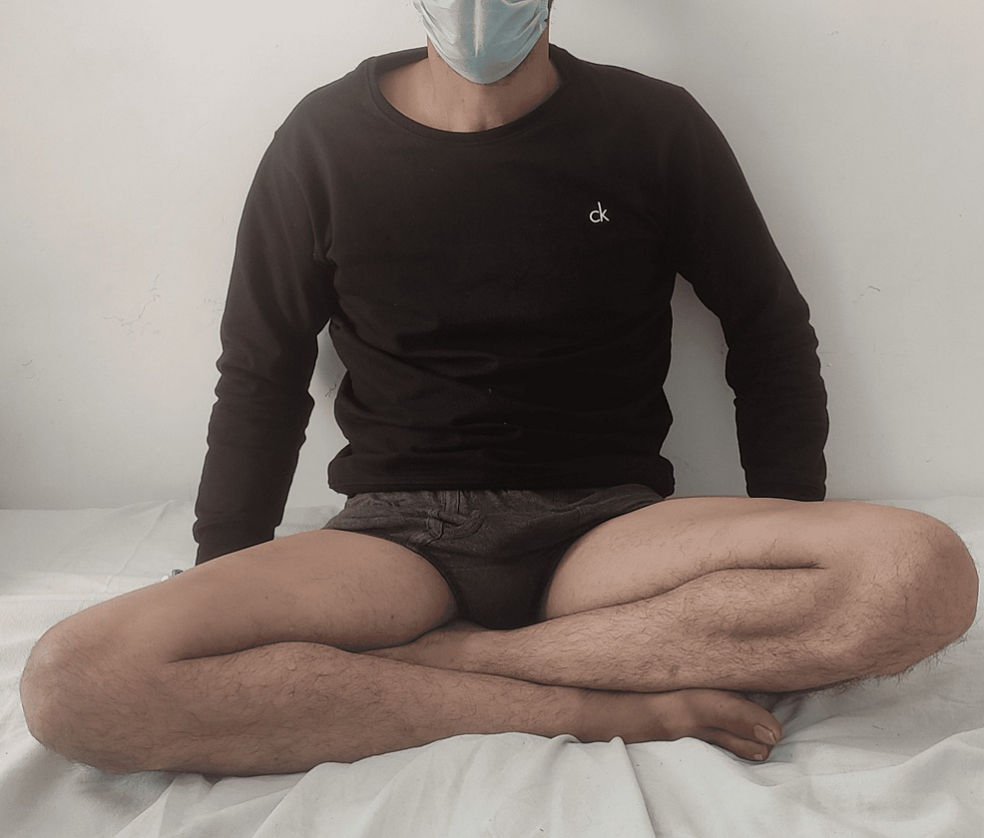
Clinical picture showing the patient sitting in a cross-legged position

**Figure 6:**
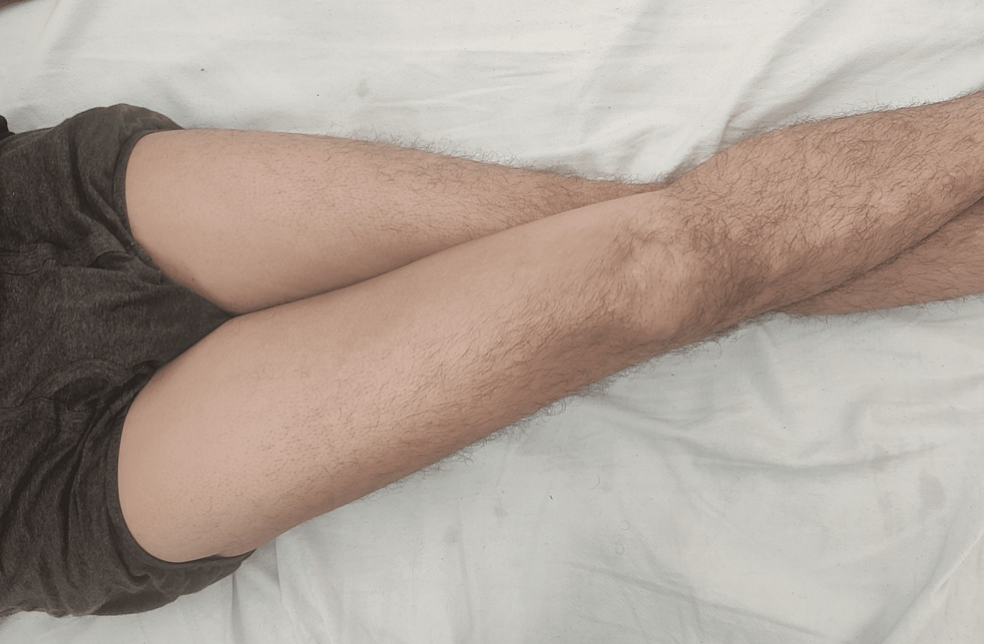
Hip adduction with the knee in an extended position Normal hip adduction ranges from zero to 35 degrees

**Figure 7:**
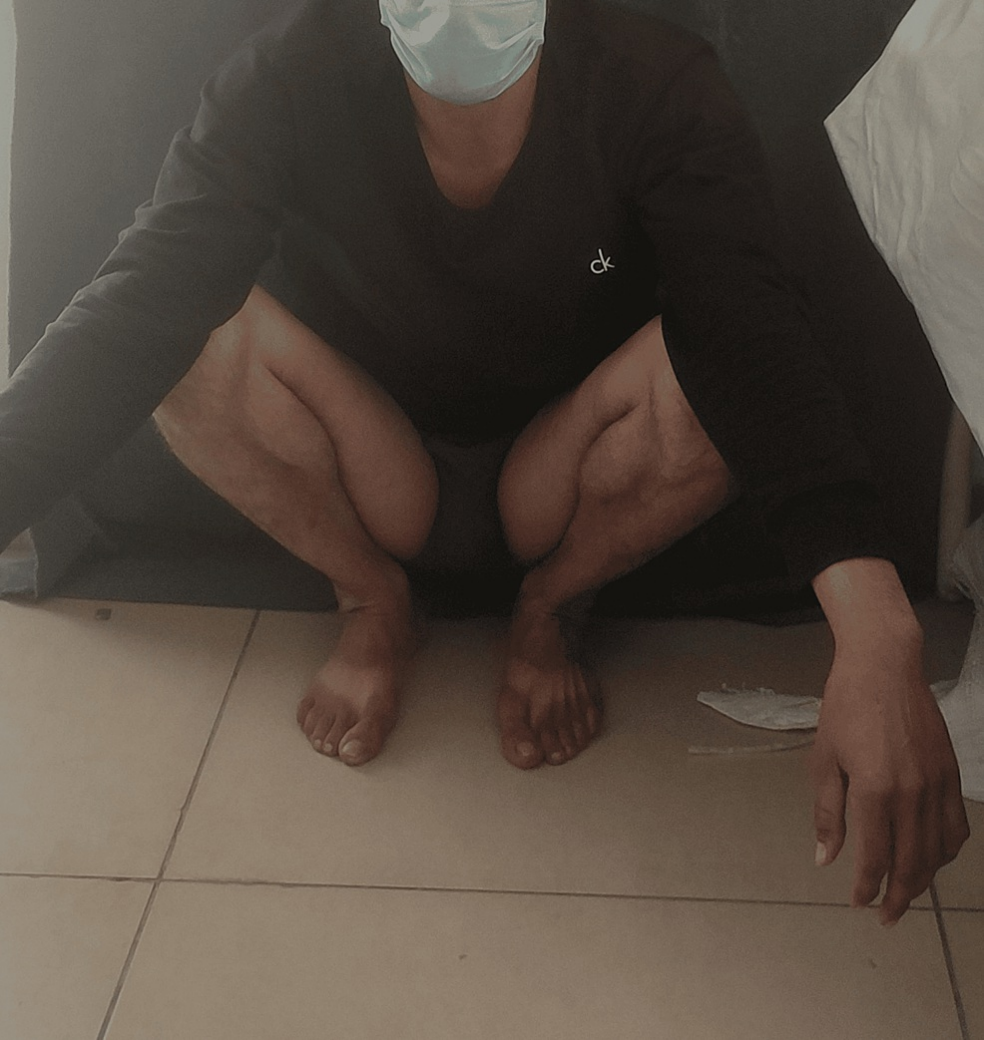
Clinical picture showing the patient in a squatting position

Eleven patients (18.66%) had diabetes, while nine (15%) had hypertension. One (1.66%) was hepatitis C virus (HCV)-positive, one (1.66%) was hepatitis B surface antigen (HBsAg)-positive, and one (1.66%) was suffering from hypothyroidism. Fracture patterns were divided into various types according to Boyd and Griffin's classification of intertrochanteric fractures and according to Seinsheimer's classification of subtrochanteric fractures. The most common intertrochanteric fracture was Boyd and Griffin's type 2 (24 patients, 40%), while the most common subtrochanteric fracture was Seinsheimer's type IIB (seven patients, 11.66%). In 52 patients (86.66%), closed reduction was achieved, while in the remaining eight patients (13.33%), fracture was opened to achieve the reduction, followed by fixation with PFNA-2. The intraoperative blood loss was quantified by the number of mops employed during the surgical procedure. Approximately 50 mL of blood loss is equivalent to one mop. The mean blood loss was 1.85 mop, resulting in a quantity of 92.66±15.27 mL. Out of the total number of patients, six (10%) needed to undergo an intraoperative blood transfusion due to their low preoperative hemoglobin levels. Two patients (3.32%) had associated shaft of femur fractures on the same side, one patient (1.66%) had medial femoral condyle fracture of the opposite side, one (1.66%) had L4 fracture without neurodeficit, and one (1.66%) had L1 and D12 fractures without neurodeficit. Both spinal fractures were managed conservatively, and weight-bearing in both of these cases was delayed. Figure 1 and Figure 2 show preoperative X-rays, and Figure 3 and Figure 4 show post-operative X-rays, along with clinical pictures of one patient who was included in the present study (Figures 5-7).

The average operating time in our study was 53.03±5.66 minutes after anesthesia. The mean duration of hospitalization was 8.08±2.56 days, with a post-operative hospital stay of 2.61±0.78 days. Nevertheless, the mean duration from injury to operation was 5.47±1.74 days. The variability in patient outcomes can be attributed to factors such as the accessibility of the operating theatre and the presence of comorbid conditions among the patients. Knee stiffness was the most common complication encountered, seen in seven patients (11.66%). Six individuals (10%) experienced a superficial wound infection as a result of wound complications, out of which two individuals were suffering from type 2 diabetes mellitus. All of these patients' illnesses resolved after undergoing wound cleansing and receiving antibiotics that were sensitive to culture. The union rate in the present study was 100%. No instances of peri-implant femoral fractures occurred after the surgery. The modified Harris hip score was used to evaluate the morphological and functional results of all patients who had undergone fracture union or were 16 weeks old. Various parameters of the modified Harris hip score are shown in Table 2. The range of motion is determined by using the modified Harris hip scoring system; 43 patients (71.66%) had a score of 5 indicating a good range of motion, while 17 patients (28.33%) had a score of 4. The modified Harris hip score had an average value of 84.78±4.31. The results were as follows: 26.66% (16 patients) were excellent, 70% (42 patients) were good, and 3.33% (two patients) were fair.

**Table 2:** Functional evaluation by the modified Harris hip score

Parameter of the modified Harris hip score		Number of patients	Percentage
Pain	None	35	58.33%
	Slight	10	16.66%
	Mild	9	15%
	Moderate	6	10%
	Marked	-	-
	Disabling	-	-
Limp	None	48	80%
	Slight	12	20%
	Moderate	-	-
	Severe	-	-
Support	None	47	78.33%
	Single cane for long walks	11	18.33%
	Single cane for most of the time	2	3.33%
	One crutch	-	-
	Two canes	-	-
	Two crutches	-	-
	Not able to walk at all	-	-
Distance walked	Unlimited	42	70%
	<1000 m	11	18.33%
	<500 m	7	3.33%
	Indoor only	-	-
	Bed and chair	-	-
Activity			
Stairs	Without banister	28	46.66%
	Using banister	23	38.33%
	In any manner	9	15%
	Unable	-	-
Putting on shoes and socks	With ease	42	70%
	With difficulty	18	30%
	Unable	-	-
Sitting	Comfortable in any chair for one hour	59	98.33%
	Comfortable in high chair for one hour	1	1.6%
	Unable to sit in any chair	-	-
Public transportation	Able to enter	59	98.33%
	Unable to enter	1	1.6%

## Discussion

There is an ongoing dispute regarding the suitable approach and the optimal implant to be employed. Each strategy (extramedullary and intramedullary implants) possesses distinct benefits and drawbacks. The current investigation examined a cohort of 60 individuals, regardless of gender, who presented with peritrochanteric fractures. The PFNA-2 is a highly efficient device for sharing intramedullary load. The advantages of PFNA-2 include smaller incisions, less blood loss, reduced operational time, and early weight-bearing. The union rate in the present study was 100%.

The study participants comprised an average of 56.56 years of age, spanning a range of 20-102 years. The mean of this study was found to be lower in comparison to previous studies. This is presumably due to the higher life expectancy in industrialized nations. With a mean age of 70 years, Nungu et al. 1993 [4] examined 15 patients ranging in age from 20 to 95 years. In their 2007 study, Jiang et al. [5] evaluated 49 patients with a mean age of 53 years and ages ranging from 22 to 78 years. According to a study by Mereddy et al. (2009) [6], the mean age of 62 patients was 78 years, with a range of 44-94 years. In their study, Loo et al. (2011) [7] assessed 62 participants, ranging in age from 22 to 99 years with an average of 74.3 years. In their 2015 study, Kumar et al. [8] examined 45 patients ranging in age from 35 to 90 years, with a mean age of 61 years. In their study, Hao et al. (2019) [9] assessed 45 patients whose ages ranged from 19 to 92 years, with an average age of 71.6 years. Similarly, Swaroop et al. (2020) [10] examined 61 individuals, with an average age of 73.39 years.

We found that males had a higher propensity to be impacted than females, which distinguishes this investigation from those of Tomás et al. (2013) [11], Li et al. (2014) [12], and Sadic et al. (2014) [13]. The gender composition of the patient population is male-dominated; this discrepancy could potentially be attributed to the higher incidence of high-energy trauma among males in comparison to females of equivalent age. In line with prior investigations conducted by Sahin et al. (2014) [14] and Loo et al. (2011) [7], our results indicated that left-side involvement was more prevalent than right-side involvement (38.33% versus 61.66%). Eighty percent of the patients experienced a trivial fall and 20% an RTA, according to research by Nungu et al. (1993) [4], whereas Mereddy et al. (2009) [6] discovered that 77% of the patients encountered a trivial fall, 18% RTA, and 5% an alternative form of injury. Our results are consistent with those of the aforementioned studies. Sadic et al. (2014) [13] discovered that 90.5% of the participants had minor falls, whereas only 35 had serious traffic accidents, 4.7% had falls from heights, and 1.5% had injuries from other sources. Li et al. (2014) [12] discovered that 71.8% of the participants had minor falls, while 28.2% had serious traffic accidents. According to Mu and Zhou (2021) [15], 71.19% had insignificant falls, while 28.81% experienced RTA. A closed reduction was achieved in 86.66% of the participants involved in our study. Comparable research has been conducted by Wang et al. (2010) [16], Li et al. (2014) [12], and Mereddy et al. (2009) [6].

The study conducted by Sahin et al. (2014) [14] reported an average surgical duration of 53 minutes, consistent with prior studies conducted by Soucanye de Landevoisin et al. (2012) [17] (47 minutes) and Mu and Zhou (2021) [15] (54.94±7.29 minutes), which reported a range of 32-96 minutes. The duration of hospital stays in our study was found to be eight days, with an average range of 5-11 days. The majority of the patients remained hospitalized immediately prior to surgery, and almost all were discharged on day 3 or 4 following their surgeries. In contrast to the findings of Sadic et al. (2014) [13], an average hospital stay of 12 days was observed, with a range of 7-19 days. In contrast, Mallya et al. (2019) [18] reported an average hospital stay of 6.64±1.4 days, with a range of 4-11 days. In a study conducted by Swaroop et al. (2020) [10], the average surgical blood loss was determined to be 110.66 mL. Similarly, Mu and Zhou (2021) [15] reported a mean surgical blood loss of 119.69±19.43 mL, which aligns with the findings of the current study. The prevalence of superficial infection among the three patients in our study, accounting for 5% of the total, aligns with prior research findings that have reported similar rates such as 4.8% in Loo et al. (2011) [7], 4.8% in Mohan et al. (2016) [19], 4.7% in Sadic et al. (2014) [13], and 4.7% globally. The average modified Harris hip score of 84.78 in our study aligns with the findings of prior research conducted by Shi et al. (2014) [20] (84.6%) and Mu and Zhou (2021) [15] (80.73). However, Li et al. (2014) [12] and Mallya et al. (2019) [18] reported mean HHS values of 81.6 and 81.6, respectively. The findings of our study have been compared with those of previous research in Table 3. The limitation of our study is the absence of a control group to compare the functional outcome of proximal femoral nail antirotation-2 (PFNA-2) in peritrochanteric fractures of the femur with other implants. A smaller sample size may be the other drawback. We recommend such prospective studies with a larger sample size to gauge the functional outcome and complications associated with this implant. Moreover, this study was conducted in a single institution, which might have restricted the diversity of patient populations.

**Table 3:** Comparison of the results of our study with previous studies

	Li et al. (2014) [12]	Kumar et al. (2015) [8]	Mohan et al. (2016) [19]	Mallya et al. (2019) [18]	Mu and Zhou (2021) [15]	Present study
Number of patients	163	45	108	37	59	60
Excellent	25%	35.5%	90%	13.52%	33.90%	26.6%
Good	56.5%	42.8%	10%	32.4%	49.15%	70%
Fair	16%	14.3%	-	18.9%	10.17%	3.33%
Poor	2.5%	7.1%	-	35%	6.78%	-

## Conclusions

Based on our analysis, it can be inferred that PFNA-2, when well-executed, is a secure and straightforward alternative implant for treating peritrochanteric fractures, because it preserves periosteal covering and provides short operative time and early mobilization. PFNA-2 provides very good outcomes in most patients with peritrochanteric fractures with minimum complication rates as compared to all other open methods and is highly recommendable.
